# Sea Sand as a Silica Source to Hydrothermally Synthesize Analcime

**DOI:** 10.3390/ma18122818

**Published:** 2025-06-16

**Authors:** Wei Xie, Hao Ma, Chuangguang Cao, Yating Wang, Yanhui Qiao, Junjiang Teng, Ning Li, Chaochao Yue

**Affiliations:** College of Chemistry, Guangdong University of Petrochemical Technology, Maoming 525000, China; wxie_gdupt@163.com (W.X.); thma@gdupt.edu.cn (H.M.); ccgcaochuang1@163.com (C.C.); wangyatingych@163.com (Y.W.); qyhmmc@gdupt.edu.cn (Y.Q.); tjjteng@gdupt.edu.cn (J.T.); liningmmc@gdupt.edu.cn (N.L.)

**Keywords:** sea sand, synthetic analcime, hydrothermal synthesis, Cu^2+^ adsorption

## Abstract

Analcime has demonstrated potential for a variety of applications in technology, especially in adsorption and heterogeneous catalysis. In this study, synthetic analcime was investigated by using sea sand as a silica source. Sea sand was first treated with HNO_3_ and NaOH. The pretreated sea sand as the silica resource and Al(NO_3_)_3_ as the aluminum source were used for the hydrothermal synthesis of analcime with different ratios of Si/Al and Na/Si. The products obtained under different conditions were characterized by X-ray diffraction. The results showed that analcime synthesized using acid-treated sea sand was mixed with other impurities, such as quartz and sodalite. Pure analcime was obtained using alkali-treated sea sand as the silica source. The analcime prepared under an optimized synthesis condition was further investigated via SEM, FT-IR, and TG. The particle size of the prepared analcime ranged from 40 to 50 μm. The adsorption ability of analcime was studied, and the Cu^2+^ adsorption process was found to follow a *pseudo*-second-order kinetic model.

## 1. Introduction

Zeolites, with their unique internal structure, are widely used as catalysts, adsorbents, and ion exchangers [1,2,3,4]. Zeolites are typically hydrated aluminosilicate minerals that can be classified as either natural or synthetic. Natural zeolites occur in volcanic and chemical sedimentary rocks [5,6]. Synthetic zeolites can be prepared using several materials containing a rich source of Si and Al [7,8]. For the synthesis of zeolites, starting material(s) or chemical(s) (e.g., kaolin, rice husk ash, and coal fly ashes) have been studied in the past few decades, although most of these have generally been considered waste materials [9,10,11,12]. Environmental problems can be reduced by using these solid waste materials as starting sources of silica and alumina. Additionally, as waste materials, the zeolites’ production cost is also lower than that when using pure chemical reagents.

Analcime has irregular channels formed from four-, six-, or eight-fold rings and can be used as an absorbent in the treatment of wastewater and heterogeneous catalysts [13,14,15]. Analcime synthesized by using metakaolin and rice husk ash has been studied to investigate the adsorption of phenol, and the adsorption capacity was found to be about 33.1 mg/g [9]. Furthermore, analcime prepared from steel slag and coal fly ash has been used to remove heavy metal ions in polluted water [16,17]. The synthesized analcime demonstrated a fantastic adsorption capacity, and the removal rate and uptake of heavy metal ions by analcime was quite rapid. The maximum adsorption was reached in the first hour of contact, although the adsorption equilibrium was reached slowly after the initial rapid adsorption [17].

Analcime is normally synthesized by using sodium silicates and aluminate. However, the preparation of analcime from silica and alumina, especially the synthetic materials, is a rather costly process. It is fascinating to design a sustainable route to synthesize analcime, making it environmentally friendly and needing fewer resources. Inexpensive aluminosilicate materials, such as coal fly ash, rice husk ash, quartz syenite, and pyrophyllite, have been used to synthesize analcime [18,19,20,21]. These materials also have the advantage of alleviating environmental pollution challenges. However, pure zeolite phases are difficult to obtain using these solid wastes as aluminosilicate sources, and this problem limits the further application of zeolites. In previous research, co-crystallizations of analcime were obtained by using kaolin in the hydrothermal synthesis of analcime; the synthesis methods were tested in the presence of different NaOH concentrations, with variable temperatures ranging from 170 °C to 210 °C and different reaction times [22,23,24]. Due to the presence of insoluble crystalline phases such as quartz and mullite in coal fly ash, the cation exchange capacity of zeolite A synthesized using coal fly ash was lower than that using the pure Si and Al source.

Compared to the other natural minerals and solid waste materials mentioned above, sand made up of the main phase of quartz has more potential as a raw material in the synthesis of different types of zeolites [25,26]. Pure zeolite Y was obtained under the optimum crystallization temperature and time. Hernandez-Palomares investigated the zeolites A, X, and P using ore sand [26]. The sand was thermally treated with NaOH at 250 and 550 °C. Sea sand is also composed of the main phase of quartz, and this resource is natural and abundant. Due to the excessive chloride content, sea sand has not been widely applied and is currently mainly used as a raw material in concrete, resulting in a low utilization value [27]. In this study, sea sand was used as a raw material for the synthesis of zeolite. It was pretreated with different methods: (1) treated with HNO_3_ to remove metals such as iron, and (2) treated with HNO_3_ and NaOH. In the second pretreatment method, the quartz in the sea sand was dissolved. The final sea sand treated with different methods was used as the silica source to synthesize analcime. Analcime mixed with quartz and sodalite was obtained using acid-pretreated sea sand. Pure analcime was synthesized using acid–alkali-treated sea sand. The particle size of the prepared analcime was 40–50 μm. Subsequently, Cu^2+^ solutions were prepared for the investigation of metal ion adsorption.

## 2. Materials and Methods

### 2.1. Sea Sand Pretreatment

**Figure 1:**
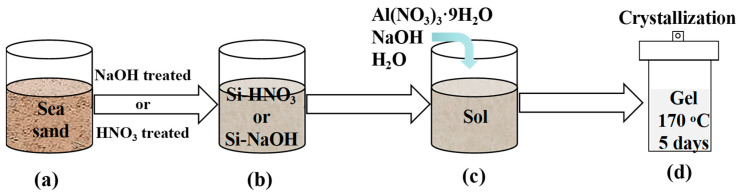
Sea sand pretreatment and analcime synthesis: (a) ground sea sand, (b) acid-treated sea sand or alkali-treated sea sand, (c) mixed gel of the synthesis of analcime, and (d) crystallization of analcime.

Before the acid or alkali pretreatment, sea sand (local beach, Maoming, China) was ground in a ball mill to produce a particle size of 60 mesh and then washed in deionized water with a mass ratio of 1 to 5. Figure 1a,b show the sea sand treatment using HNO_3_ or NaOH and analcime synthesis [25].

Acid treatment (Figure 1a,b): Briefly, 10 g of sea sand from the local beach was mixed with 500 g of 5 M HNO_3_ (65%, Macklin, Shanghai, China) at 30 °C. After 10 h of stirring, the solid was filtered and washed to pH = 7. After drying in the oven for 12 h at 80 °C, the washed solid was calcined at 500 °C for 6 h. The calcined sample was named Si-5HNO_3_. Sea sand was washed in 3 M HNO_3_ and 4 M HNO_3_. After drying and calcination as in the above methods, the obtained samples were named Si-3HNO_3_ and Si-4HNO_3_, respectively.

Alkali treatment: To remove the metal oxide in the sea sand, 10 g of sea sand from the beach was first mixed with 100 g of 1 M HNO_3_ at 30 °C for 5 h. After acid washing, the sea sand was stirred in 20 g of 10 M NaOH (99.9%, Macklin, Shanghai, China) at 30 °C for 5 h [26]. After drying the washed sea sand at 80 °C, the washed solid was calcined at 500 °C for 6 h. Then, 10 g of the calcined sample was further mixed with 30 g of water at 30 °C for 5 h of stirring. The pH of the mixed solution was adjusted to 7 by using 5 M HCl. Si-NaOH was obtained after filtering and drying.

### 2.2. Preparation of Analcime

Figure 1c,d show the synthesis of analcime using Si-HNO_3_ or Si-NaOH. The initial gels for the synthesis of analcime-type zeolites were prepared using pretreated sea sand, Al(NO_3_)_3_·9H_2_O (99.9%, Aladdin, Shanghai, China), NaOH, and distilled water. In a typical synthesis, Al(NO_3_)_3_·9H_2_O was dissolved in an aqueous alkali solution under stirring for 30 min, and Si-HNO_3_ or Si-NaOH was subsequently added slowly. The resulting mixture was homogenized under stirring for an additional 3 h. The gel had the following molar ratio: 6SiO_2_:xAl_2_O_3_:yNaOH:60H_2_O, in which x is from 0.75 to 1.5 and y is from 6 to 60. The mixture was transferred to a 50 mL Teflon-lined stainless steel autoclave and treated at 170 °C for 5 days under static conditions. The solid products were filtered and washed with distilled water until the filtrate pH was 7. The recovered products were dried at 80 °C for 12 h.

### 2.3. Adsorption Experiments

The adsorption performance of analcime was evaluated using batch experiments. A Cu^2+^ standard solution with an initial concentration of 300 mg/L was prepared, and the pH of the solution was adjusted to 3 with 6 M nitric acid. Then, 0.1 g of the prepared analcime as the absorbent and 100 mL of the Cu^2+^ solution were mixed and shaken at a speed of 100 rpm to reach equilibrium at 25 °C. The contact time between the absorbent and the Cu^2+^ solution was set at 2, 4, 6, 8, 10, 15, 30, 60, and 90 min. Moreover, concentrations of 100, 150, 200, 250, and 300 mg/L Cu^2+^ solutions were also prepared to explore the adsorption capacity of the adsorbent to Cu^2+^. Reaction products were filtered, and the filtration was tested by ICP-OES.

The experiment data were used to model adsorption kinetics by fitting them to the *pseudo*-first-order kinetic model (Equation (1)) and the *pseudo*-second-order kinetic model (Equation (2)) [28,29]:

(1) $$ln(q_{e}-q_{t})=lnq_{e}-K_{1}t$$

(2) $$\frac{1}{q_{t}}=\frac{1}{{q_{e}^{2}K}_{2}}+\frac{t}{q_{e}}$$

where *q_e_* is the adsorption capacity at equilibrium (mg/g), *q_t_* is the amount of Cu^2+^ adsorbed at time t (mg/g), and K_1_ and K_2_ are the rate constants of the *pseudo*-first-order kinetic model and *pseudo*-second-order kinetic model, respectively.

### 2.4. Characterization Techniques

Samples were characterized with X-ray diffraction (XRD, Ultima IV, Rigaku, Tokyo, Japan) using a Cu Ka X-ray generated with a current of 40 mA and a potential of 40 kV. The scan range was set from 10° to 50°, and the scan speed was 5°/min. The scanning electron microscopy (SEM) images were taken on a JSM-6510LV and Regulus 8220 (Tokyo, Japan) instruments operating at 5 kV, and the samples were coated with gold to increase conductivity. Chemical compositions were determined on a Regulus 8220 microscope by energy spectrum analysis (EDX). The specific surface area of the samples was obtained through N_2_ physisorption measurements. The samples were first pretreated at 200 °C for 2 h under vacuum. The analysis was carried out using Micromeritics equipment, ASAP 2460 (Micromeritics, Norcross, GA, USA). The specific surface area was determined by the multi-point BET (Brunauer–Emmett–Teller) method. The concentration of Cu^2+^ was tested by inductively coupled plasma optical emission spectroscopy (ICP-OES) on a Thermo Fisher iCAP 7600 (Waltham, MA, USA). Fourier-transform infrared (FT-IR) spectroscopy was investigated (Nicolet 6700, Thermo Scientific, Waltham, MA, USA) with a resolution of 2 cm^−1^ using the KBr method. The TG profile was performed on NETZSCH STA499F3 (Bavaria, Germany) by using a reactive O_2_ flow of 40 mL/min and a protective N_2_ flow of 20 mL/min.

## 3. Results

The elemental components of sea sand obtained under EDX are shown in Table 1. The major elements in the treated sea sand were Si, O, Cl, and Na. After acid or alkali–acid treatment, the Si content increased from about 22.59% to 32.27–33.46%. The quantities of Al and K in the sea sand subject to acid treatment or alkali–acid treatment were about 1–2% and 0.6–0.7%, respectively. The amount of Fe was about 0.15% in the sea sand without treatment, and Fe was removed after acid or alkali–acid treatment [30]. This caused the color of the treated sea sand to become considerably closer to white, differing from the brown color of the original sea sand.

**Figure 2:**
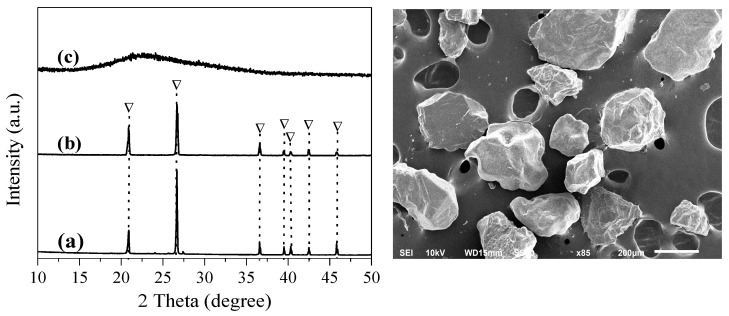
XRD patterns of (a) sea sand, (b) acid-treated sea sand, and (c) alkali-treated sea sand, and an SEM image of the sea sand. ▽: Quartz.

Figure 2 shows the XRD patterns of the original and the pretreated sea sand. In Figure 2a, the pattern of the original sea sand possesses some diffraction peaks, indicating that the material is mainly composed of quartz (SiO_2_, ICSD 85-1054), which is matched with the diffraction peaks at 20.8°, 26.7°, 36.6°, 39.5°, 40.4°, 42.5°, and 45.8° (d values at 4.2550 Å, 3.3435 Å, 2.4569 Å, 2.2815 Å, 2.2361 Å, 2.1277 Å, and 1.9799 Å, respectively). The SEM image shows that the sea sand has irregular shapes of different sizes. The quartz phase was not destroyed after acid treatment (Figure 2b). After alkali treatment (Figure 2c), the quartz was dissolved, and only a halo pattern was observed.

**Figure 3:**
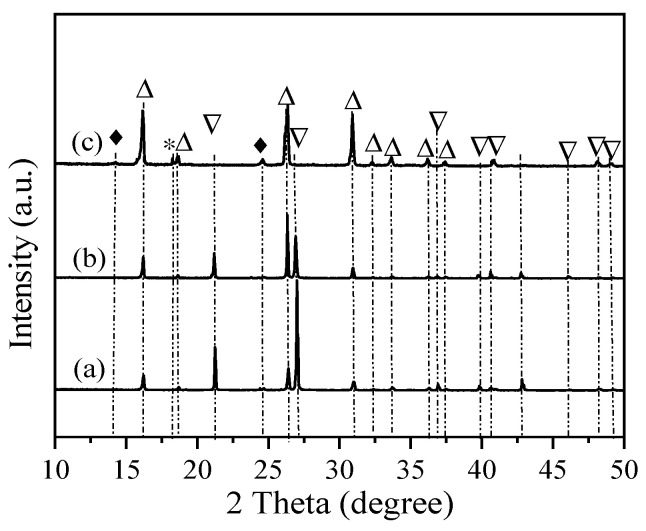
XRD patterns of the single-factor experiment, showing analcime synthesized using acid-treated sea sand with a gel composition of 6SiO_2_:1Al_2_O_3_:6NaOH:60H_2_O: (a) 1 day, (b) 2 days, and (c) 3 days. △: Analcime; ▽: quartz; ♦: sodalite; *: Al(OH)_3_.

The synthesis times were investigated for the synthesis of analcime using acid-treated sea sand with a gel ratio of Si/Al = 3 and Na/Al = 1. As shown in Figure 3a, after 1 day of reaction, the main phases were analcime and quartz. When the synthesis time was 3 days, the diffraction intensity of the quartz peaks clearly gradually decreased, and the analcime was stronger (Figure 3b). In Figure 3c, a well-developed analcime became the dominant product when the synthesis time was 5 days.

**Figure 4:**
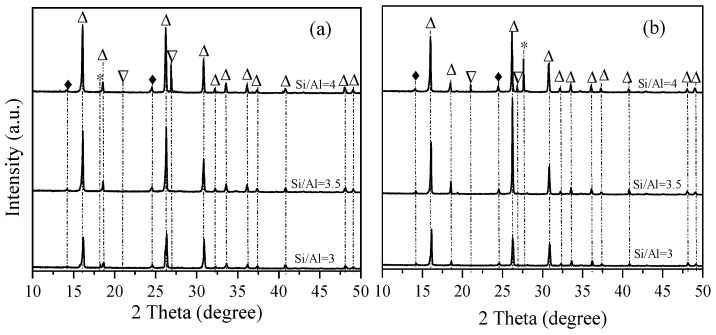
XRD patterns of analcime synthesized from sea sand pretreated with different concentrations of HNO_3_: (a) 3 M HNO_3_, (b) 4 M HNO_3_. △: Analcime; ▽: quartz; ♦: sodalite; *: Al(OH)_3_.

The prepared Si-HNO_3_ was used to synthesize analcime. The successful synthesis of analcime-type zeolites was performed at 170 °C for 5 days, using gel with an initial composition of 6SiO_2_:xAl_2_O_3_:6NaOH:60H_2_O. Si-HNO_3_ prepared with different concentrations of HNO_3_ was used as the silica source to synthesize analcime. The effect of the Si/Al ratio on the zeolitization of analcime is shown in Figure 4, using Si-HNO_3_ prepared with 3M HNO_3_. Sodalite (ICDD 37-0476) was observed with a ratio of Si/Al = 3, and the characteristic peaks are located at 14.2° and 24.7° (d values at 6.2800 Å and 3.624 Å). Upon increasing the ratio of Si/Al, sodalite was still formed, and quartz was observed with peaks at 20.9° and 26.7° (d values at 4.2550 Å, 3.3435 Å). A similar trend was observed by using Si-HNO_3_ prepared with 4M HNO_3_. The results suggest that the synthesized products contain analcime (ICDD 41-1478) as the major constituent phase, with the ratio of Si/Al between 3 and 4. The characteristic peaks of analcime in patterns are located at 15.8°, 18.3°, 24.3°, 25.9°, 30.5°, 31.9°, 33.3°, 35.9°, 37.1°, 40.6°, 44.8°, 46.9°, 47.8°, and 48.8° (d values at 5.5901 Å, 4.8438 Å, 3.6671 Å, 3.4254 Å, 2.9209 Å, 2.7970 Å, 2.6875 Å, 2.5007 Å, 2.4231 Å, 2.2223 Å, 2.0204 Å, 1.9375 Å, 1.9012 Å, and 1.8653 Å) [31]. The results are consistent with the theoretical Si/Al ratio for analcime crystallization of about 2; for natural analcime, the ratio of Si/Al is between 3.6 and 5.6 [32]. Furthermore, the influence of Si/Al was evaluated in the conventional synthesis of analcime using silica aerosil [13]. Although high-crystallinity analcime was obtained across a wide range of Si/Al ratios, from 20 to 100, no analcime was formed with the ratio of Si/Al = 10; however, amorphous materials were formed.

**Figure 5:**
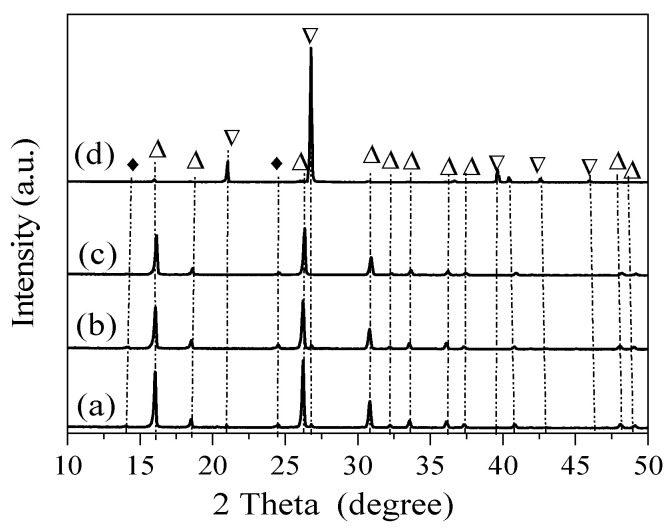
XRD patterns of analcime synthesized by using Si-HNO_3_ with different ratios of Na/Si: (a) Na/Si = 1, (b) Na/Si = 2, (c) Na/Si = 3, (d) Na/Si = 5. Si-HNO_3_: sea sand pretreated with 5 M HNO_3_. △: Analcime; ▽: quartz; ♦: sodalite.

Sea sand pretreated with 5 M HNO_3_ was also used to synthesize analcime at a gel ratio of Si/Al = 3. As shown in Figure 5, analcime mixed with minor sodalite and quartz was obtained with a ratio of Na/Si = 1 and 2. From the XRD patterns of the sample prepared with a ratio of Na/Si = 3, the mixed phases quartz and sodalite disappeared, and only analcime with intense diffraction peaks at 15.8°, 25.9°, and 30.5° was formed [17]. By increasing the ratio of NaOH/Si to 5, the crystalline phase was transformed into quartz mixed with sodalite and analcime. These results reveal that a high NaOH concentration is disadvantageous to the formation of analcime [20]. Therefore, NaOH/Si = 3 was taken as the optimum ratio to synthesize analcime.

**Figure 6:**
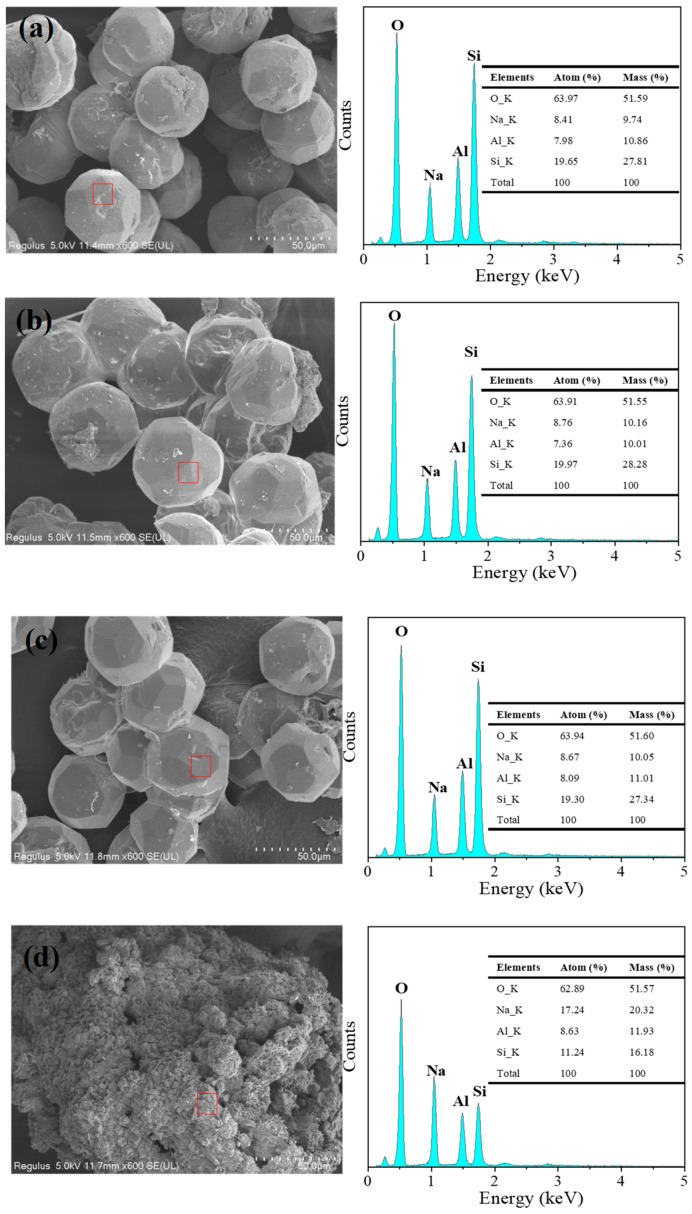
SEM-EDX spectra of analcime synthesized by using Si-HNO_3_ with different ratios of Na/Si: (a) Na/Si = 1, (b) Na/Si = 2, (c) Na/Si = 3, (d) Na/Si = 5. Red square: micro-area for EDX.

The morphology of the zeolitic products obtained with a NaOH/Si mass ratio of 1 to 5 is shown in Figure 6. With a Na/Si ratio of 1, well-developed analcime was observed in the scanned sample (Figure 6a). Upon increasing the ratio of Na/Si to 2 and 3, analcime with the characteristic isometric trapezohedral morphology was observed (Figure 6b,c), though some of its trapezohedral structure was destroyed. In addition, some crystals exhibited intergrowths and fractures. The particle size ranged from 40 to 50 μm, and smaller granules emerged on the surface of the analcime crystals. According to previous research, the formation of granules is due to the nuclei first being converted by the amorphous gel [33,34,35]. In Figure 6a,b, a blocky structure was also observed, probably from the undissolved sea sand. The morphologies of the obtained analcime in this study are similar to those reported in studies in which kaolin, rice husk ash, and clinker were used as the raw material [9,11]. The particle size of the analcime synthesized by using sea sand is larger than that reported when using kaolin, rice husk, or other raw materials [9,11]. This is because the analcime synthesized using sea sand has a longer hydrothermal time. By increasing the ratio of Na/Si to 5, as shown in Figure 6d, the fine particles agglomerated into larger particles. In addition, the chemical composition of the obtained products was analyzed by EDX. As shown in Figure 6a–c, analcime as the main phase presented a structural Na:Si:Al molar ratio of about 1:2:1. In Figure 6d, the Na:Si:Al molar ratio is about 3:2:1.5 in the mixed phase prepared at a high gel ratio of Na/Si = 5.

**Figure 7:**
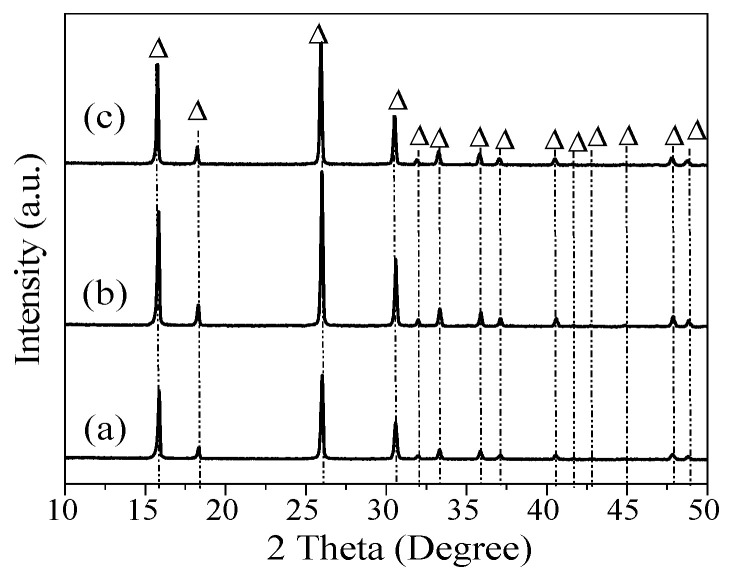
XRD patterns of analcime synthesized by using Si-NaOH with different ratios of Si/Al: (a) 3, (b) 3.5, (c) 4. △: Analcime.

**Figure 8:**
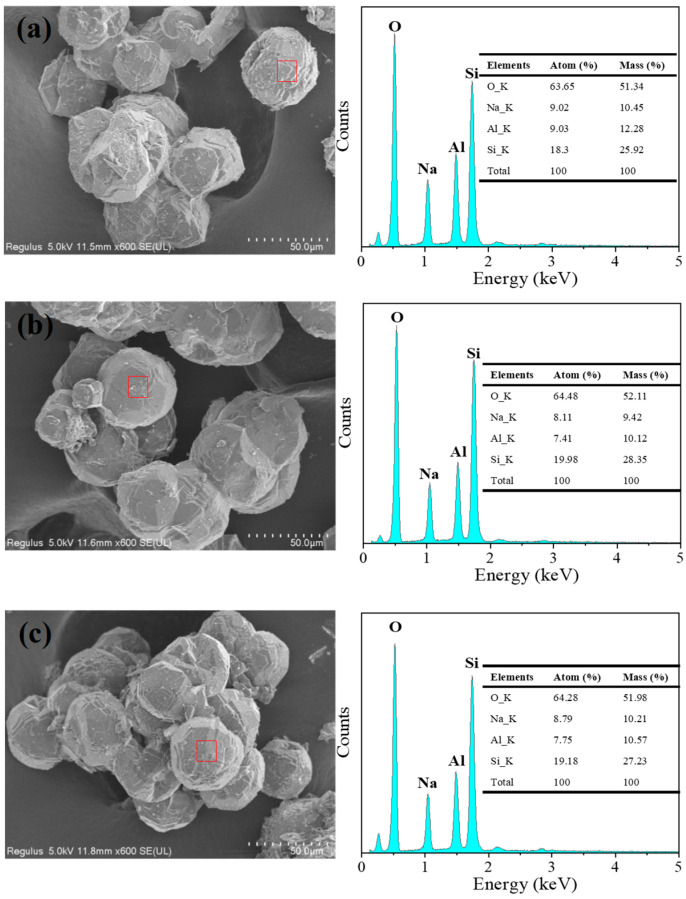
SEM-EDX spectra of analcime synthesized by using Si-NaOH with different ratios of Si/Al: (a) 3, (b) 3.5, (c) 4. Red square: micro-area for EDX.

Although the sea sand was treated with different concentrations of HNO_3_, quartz was formed in the synthesis of analcime at a higher ratio of Si to Al. In addition, a small amount of sodalite was constantly present in the synthesized analcime. These results were similar to the previous investigations into the synthesis of analcime from minerals or solid wastes, and one of the problems is the difficulty in synthesizing the pure analcime phase [36,37]. To synthesize pure analcime, sea sand was further pretreated with acid and alkali in this study. The acid- and alkali-treated sea sand, Si-NaOH, was used to synthesize analcime with different ratios of Si/Al. From the XRD patterns shown in Figure 7, we can observe that pure analcime was obtained with a wide range of Si/Al ratios. Compared to Si-HNO_3_ as the silica source, pure analcime was obtained with a ratio of Si/Al = 3–4 when using Si-NaOH as the silica source. Figure 8 shows that the morphology of the samples synthesized by Si-NaOH is the same as the analcime synthesized by Si-HNO_3_, and the particle size is about 50 μm. The synthesis of analcime in this study demonstrates similar results to that reported by Kohoutkova [36]. Compared with the synthesis of analcime using acid-treated sea sand as the silica source, pure analcime using alkali-treated sea sand was obtained with a different ratio of Si/Al = 3–4. The chemical composition of analcime synthesized using Si-NaOH as the silica source is similar to the analcime synthesized using Si-HNO_3_, and the structural Na:Si:Al molar ratio is also about 1:2:1.

**Figure 9:**
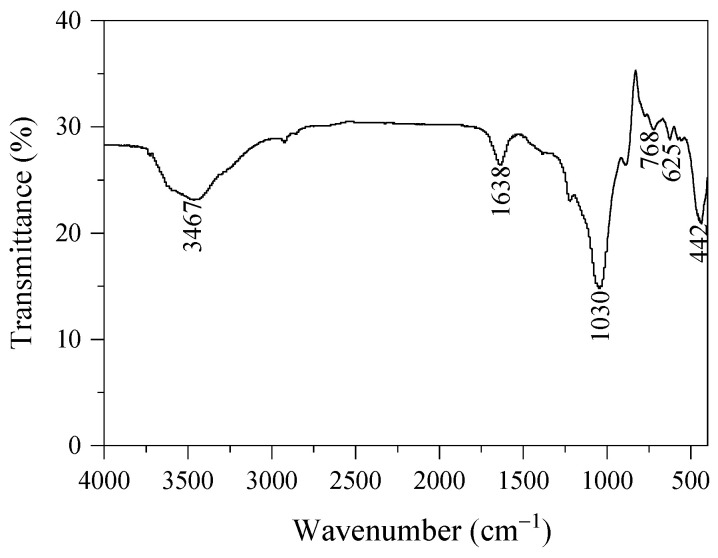
IR spectrum of analcime synthesized by using Si-NaOH with the ratio of Si/Al = 3.

Due to the similarities in structure and morphology of the prepared analcime samples, analcime prepared using Si-NaOH with the ratio of Si/Al = 3 was used to provide a further characterization. The structure of the analcime sample was investigated using the infrared spectrum. In Figure 9, the FT-IR spectra of the as-synthesized sample present bands at 3467 cm^−1^, 1638 cm^−1^, 1030 cm^−1^, 768 cm^−1^, 625 cm^−1^, and 442 cm^−1^, similar to those reported for analcime synthesized using kaolin and husk ash [11,14,37,38]. The bands at 3467 cm^−1^ and 1638 cm^−1^ are assigned to the stretching vibration and bending vibration of O–H bonds generated from the absorbed water or hydroxyl group [14]. In particular, the bands at 3467 cm^−1^ and 1638 cm^−1^ are associated with the asymmetric stretching mode of water coordinated with the edges of the channels and the zeolitic water in the channels of the zeolite [37]. The band appearing at 1030 cm^−1^ is assigned to the stretching vibration of T-O (T = Si, Al) in the asymmetric tetrahedron [TO_4_], which is the basic structure of zeolite [38]. The bands at 768 cm^−1^ and 625 cm^−1^ belong to T-O (T = Si, Al) symmetric stretching vibrations [38]. The band at 442 cm^−1^ is related to the T-O-T (T = Si, Al) bending vibration [11].

Table 2 shows that all the analcime prepared with Si-NaOH had a low specific surface area around 0.7 m^2^/g, indicating the formation of a low-porosity material. The low surface area of the analcime in this study is similar to the synthetic analcime formed using silica aerosil [13]. However, the surface area in this study is lower than that reported by Sakizci [32].

**Figure 10:**
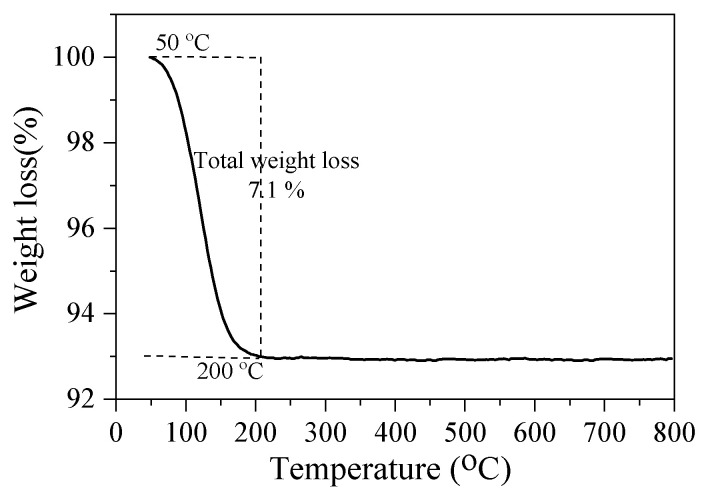
TG analysis of analcime synthesized by using Si-NaOH with the ratio of Si/Al = 3.

Figure 10 shows the thermogravimetric analysis of analcime. In particular, one well-defined dehydration step can be observed before 200 °C, and this dehydration peak corresponds to the removal of the physically absorbed water [23]. This dehydration process is different from the previous reports, in which two-step dehydration processes at 140 and 415 °C were observed [11,14]. No occluding water was found in the channel of the analcime prepared using sea sand, and the total weight loss was only 7.1%, which was lower than the previous reports.

**Figure 11:**
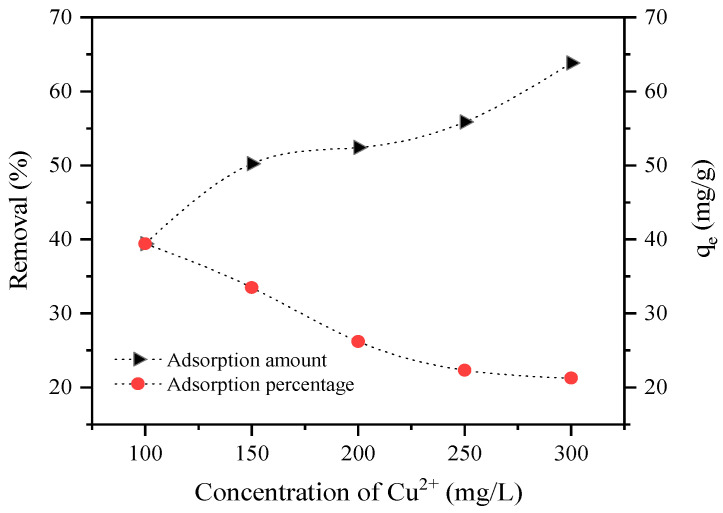
The effect of the initial Cu^2+^ concentration.

The initial solution concentration was an important factor affecting the adsorption capacity. A total of 0.1 g of analcime was added to 100 mL of 100–300 mg/L solution, and the result indicated that the adsorption capacity reached 39.4–64.8 mg/g, as shown in Figure 11. The removal of Cu^2+^ by analcime decreased with increasing initial metal concentration. This was due to the high initial concentration of the metal ions that were adsorbed at the available sites, resulting in more Cu^2+^ left unabsorbed in the solution at higher concentration levels [14]. Upon increasing the initial Cu^2+^ concentration, the driving force overcame the mass transfer resistance for metal ion transport between the solution and the surface of the analcime. Therefore, an increase in the metal adsorption capacity with increasing initial metal concentration was observed in Figure 11 [39].

**Figure 12:**
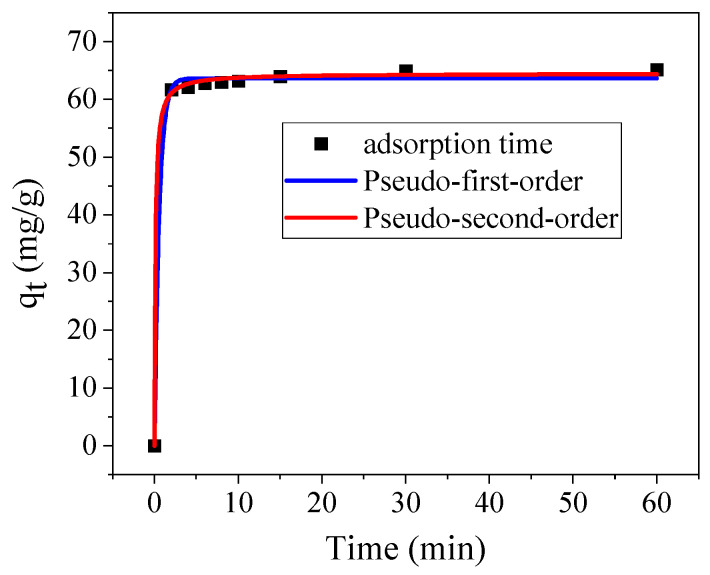
Cu^2+^ adsorption curve of the analcime.

The adsorption capacity of analcime was investigated, and the results are plotted in Figure 12. At the beginning of contact between the Cu^2+^ solution and analcime, the more active site in the analcime resulted in the fastest adsorption [17]. After a contact time of about 5 min, the adsorption equilibrium was generally reached. The maximum adsorption capacity of the prepared analcime for Cu^2+^ was 65.1 mg/g. This result displayed a similar adsorption capacity to that reported in other research studies investigating analcime synthesized using fly ash and coal gangue [14,39]. The relationship between the adsorption rate and capacity was further studied by using the adsorption kinetics. The diffusion coefficient was calculated by fitting kinetics. The corresponding results listed in Table 3 demonstrate that the correlation coefficient R_2_^2^ was higher than R_1_^2^. The maximum adsorption capacities of *pseudo*-first-order kinetics and *pseudo*-second-order kinetics were 63.64 mg/g and 64.5 mg/g, respectively, indicating that the *pseudo*-second-order kinetic model was more suitable. The results demonstrate that the adsorption of Cu^2+^ by analcime prepared with sea sand was through chemisorption [17].

Due to its widespread application in selective adsorption in wastewater treatment and heterogeneous catalysis, analcime is the most frequently used zeolite [17,40]. Due to its controllable adsorption performance, synthetic analcime has more advantages in industrial applications than natural analcime. For this reason, recent research has been more interested in the synthesis of this material using different sources of silica and alumina, particularly low-cost raw materials. The price of analcime prepared using sea sand was studied, and Table 4 shows the raw materials cost of 1 kg of analcime. The price of raw materials was taken from the chemical industry website (Guidechem). In Table 4, the raw materials cost per kilogram of analcime was about 17.726 to 21.282 USD/kg. For the industrial production of zeolites, the raw materials cost accounts for about 50% of the total manufacturing cost [41]. According to this estimation, the total manufacturing cost was about 35.464 to 42.564 USD/kg. While the manufacturing cost per kilogram is much higher than for natural analcime, it is about a 25% reduction over the cost of commercial synthetic analcime. From an economic point of view, these results suggest that utilizing sea sand as a silica source for the synthesis of analcime is a sustainable and cost-competitive process.

## 4. Discussion

Due to the nature of the silica source commonly used in the synthesis of analcime and the impurities present in it, it is difficult to prepare pure products, especially using geology and mineral resources [9,42]. In this study, sea sand was pretreated to remove metallic impurities and water-soluble salts, reducing the effect on the synthesis of analcime. When using acid-treated sea sand as the silica source, analcime and quartz were present in the obtained products. The peculiar peaks of quartz were reduced by increasing the synthesis time from 1 day to 5 days, with corresponding increases in the amounts of analcime [43].

The effect of alkalinity was investigated to improve the phase purity. In the case of a low OH^–^ concentration, analcime mixed with a trace amount of sodalite was observed. Moreover, quartz was the dominant phase, with a high OH concentration (NaOH/Si to 5). A large amount of alkali added was not favorable for the formation of analcime when using Si-HNO_3_. Additionally, the obtained analcime in this study had a consistent particle size and a regular shape. This result is the same as in some report works in the past [13]. Compared to acid-treated sea sand as the silica source, pure analcime phases were obtained using alkali-treated sea sand. This demonstrated that the silica source with different silica species led to the formation of multiple zeolite phases [44]. The element analysis showed that the Na/Si/Al mole ratio of the synthesized analcime in this study was about 1:2:1, which is similar to that of the synthetic analcime using kaolinitic rock [37].

The prepared analcime in this study had a low surface area of around 0.7 m^2^/g. The possible reason for this is that it is difficult for N_2_ with a larger atomic radius to enter the micropores of synthesized analcime [14]. Additionally, the low surface area could be attributed to the high crystallinity of these materials. Studies on the rate of uptake of Cu^2+^ by the synthesized analcime have indicated that the process was quite rapid, and the maximum adsorption occurred within the first 5 min of contact. The possible reason for this is that no water was occluded in the micropores of the synthesized analcime, and Cu^2+^ in these solutions could be quickly adsorbed. After the rapid initial adsorption, the adsorption slowly and gradually reached equilibrium, and saturation was reached in 40 and 50 min. These results show that synthesized analcime using sea sand is an effective adsorbent for Cu^2+^ removal from aqueous solutions.

Furthermore, the synthesis of analcime using sea sand as the silica source was evaluated in terms of the economic aspect. The results indicate that sea sand as a silica source is a cost-effective and available resource, making it a low-cost resource for the manufacture of high-value-added zeolite.

## 5. Conclusions

Analcime zeolite was successfully prepared by using sea sand as the silica source. Sea sand was pretreated with HNO_3_ and NaOH to remove the impurity phase. When the acid-treated sea sand was used as the silica source, analcime with high crystallinity (crystallinity of 87.36%) was obtained with a mole ratio of Si/Al = 3 and Na/Al = 3, while containing a small amount of sodalite and quartz impurities. When alkali-treated sand was used as the silica source, pure analcime was obtained with a Si/Al mole ratio of 3–4 and a Na/Al mole ratio of 3. The obtained analcime had an isometric trapezohedral morphology and a particle size ranging from 40 to 50 μm. The adsorption kinetics studies indicated that the Cu^2+^ adsorption process for analcime follows the *pseudo*-second-order kinetic model, and the adsorption of Cu^2+^ takes place through chemisorption. According to the estimation of the price of the synthesized analcime in this study, the cost can be significantly reduced by using sea sand as the silica source compared to commercial synthetic analcime. This study demonstrates a potential method for the synthesis of analcime using naturally abundant sea sand. Although developing coasts may have potential environmental impacts on marine life and coastal erosion, these impacts can be addressed through technological innovation, balancing the need for coastal development with ecological safety thresholds.

## Figures and Tables

**Table 1:** Element components of sea sand, acid-leached sea sand, and base-acid-treated sea sand (wt.%).

Element	Sea Sand	Acid-Treated	Alkali-Treated
O	42.03	42.56	50.54
Si	22.59	32.27	33.46
Cl	18.30	14.80	4.96
Na	14.41	8.72	8.24
Al	1.37	1.05	2.08
K	1.15	0.60	0.72
Fe	0.15	0	0

**Table 2:** Specific surface areas of different samples.

Sample	Si/Al Ratio	Specific Surface Area (m^2^/g)
Analcime, prepared from alkali-treated sea sand as silica source	3	0.69
	3.5	0.71
	4	0.67

**Table 3:** Fitting parameters of *pseudo*-first-order and *pseudo*-second-order adsorption kinetics.

*pseudo*-First-Order			*pseudo*-Second-Order		
K_1_ (1/min)	R_1_^2^	Q_e_ (mg/g)	K_1_ (1/min)	R_2_^2^	Q_e_ (mg/g)
1.7215	99.558	63.64	0.1353	99.832	64.50

**Table 4:** Estimated costs of production of 1 kg of analcime.

	Acid Treatment			Alkali Treatment		
	Dosage	Unit Cost (USD)	Total (USD)	Dosage	Unit Cost (USD)	Total (USD)
Sea sand	1.1 kg	0.02	0.022	1.1 kg	0.02	0.022
HNO_3_ (65 wt.%)	19.3 kg	0.2	3.86	-	-	-
NaOH	0.7 kg	0.38	0.76	1.3 kg	0.38	1.064
Al(NO_3_)_3_	2.08 kg	8	16.64	2.08 kg	8	16.64
Total	-	-	21.282	-	-	17.726

## Data Availability

The raw data supporting the conclusions of this article will be made available by the authors upon request.
